# Effects of experimental lead exposure on physiological indices of nestling great tits *Parus major*: haematocrit and heterophile-to-lymphocyte ratio

**DOI:** 10.1093/conphys/coz067

**Published:** 2019-10-28

**Authors:** Marcin Markowski, Adam Kaliński, Mirosława Bańbura, Michał Glądalski, Jarosław Wawrzyniak, Joanna Skwarska, Jerzy Bańbura

**Affiliations:** 1 Department of Experimental Zoology and Evolutionary Biology, Faculty of Biology and Environmental Protection, University of Łódź, Banacha 12/16, 90-237 Łódź, Poland; 2 Museum of Natural History, Faculty of Biology and Environmental Protection, University of Łódź, Kilińskiego 101, 90-011 Łódź, Poland

**Keywords:** Biomarkers, experimental exposure, haematocrit variation, heterophil/lymphocyte ratio, lead intoxication

## Abstract

Lead (Pb) is recognized as one of the most toxic trace elements that can induce a wide range of negative health effects in wildlife. Because the investigation of basic environment-sensitive physiological indicators is easy to perform in wild birds, they have been considered as valuable bioindicators of lead contamination. The harmfulness of lead poisoning can depend on the type of exposure, and in most studies of birds, an effect of chronic lead exposition has been considered. In this study, we investigated whether a single exposure to specific doses of lead affected such physiological indices as haematocrit and the heterophil-to-lymphocyte ratio (H/L ratio). For this purpose, we conducted an experiment based on intentional lead supplementation, with the use of two different doses, applied to randomly chosen great tit (*Parus major*) nestlings from randomly selected broods. A few days after the exposure (when the nestlings were 15 days old), we determined haematocrit and the H/L ratio as potentially sensitive physiological indicators of lead intoxication. We found that the treatment with lead resulted in a significant decline in haematocrit level. In addition, we found that the age when lead exposure occurred can be considered as an important factor influencing haematocrit variation. A decrease in haematocrit was observed on consecutive days of nestling life. In contrast, the H/L ratio did not show any significant response to lead exposure. These results clearly show that the assessment of haematocrit level for nestling great tits can provide a simple and rapid method of indirect evaluation of physiological effects of lead intoxication caused by a single exposure.

## Teaser

This study applies a field experimental approach to an issue of single lead exposures and their effects on specific haematologial indices in nestling songbirds. We used nestling great tits *Parus major* as a model and showed that higher lead exposure was associated with significant haematocrit decline. In contrast, the assessment of the H/L ratio revealed no significant effect of a single lead exposure.

## Introduction

Heavy metal contamination is a well-known phenomenon which notably concerns the urbanized and industrialized areas of the world. Undoubtedly, this is related to the growth of the human population observed on a global scale since the mid-19th century and, consequently, to the ongoing intensive development of industry (Swaileh and Sansur, 2006; Markowski *et al*., 2014). Much attention has been paid to lead (Pb), as it has long been recognized as one of the most toxic trace elements for various groups of organisms (Burger, 1998; Kålås *et al*., 2000; Danadevi *et al*., 2003; Roux and Marra, 2007). Nowadays, its distribution and abundance in the environment is primarily associated with widely understood human activity, mostly industrial, but also with transport, hunting and angling. Lead is commonly used in the production of batteries, pigments and paints, ammunition, some parts of fishing tackle and lead-glazed earthenware as well as an anti-knock additive for aviation fuel combust in piston engine aircraft (De Francisco *et al*., 2003; Katavolos *et al*., 2007; Haig *et al*., 2014; Perrault *et al*., 2017). In urbanized areas which seem to be the most exposed, the most common sources of lead are by-products of combustion processes of fossil fuels used in energy and metallurgic industry (Hoff Brait and Antoniosi Filho, 2011; Chatelain *et al*., 2016).

Being a common environmental pollutant, lead remains one of the most frequently studied trace elements, as it can exert serious negative effects on birds (Dauwe *et al*., 2000; Burger and Gochfeld, 2005; Roux and Marra, 2007; Markowski *et al*., 2013; Ruuskanen *et al*., 2015). Consequences of lead intoxication for birds can range from slight interruption of many physiological and biochemical processes to alterations in behaviour or substantial disorders of meaningful organs and systems, which in extreme cases may even cause lethal effects, depending on the level of exposition (De Francisco *et al*., 2003; Roux and Marra, 2007; Holt and Miller, 2011; Haig *et al*., 2014; Provencher *et al*., 2016). For instance, there are relatively well-documented cases of lead poisoning in waterfowl exposed to contaminated food, containing lead shot pellets or lead fishing sinkers (Scheuhammer and Norris, 1996; Pain, 1996; Fisher *et al*., 2006; Ancora *et al*., 2008; Hernández and Margalida, 2009; Eeva *et al*., 2014; Ferreyra *et al*., 2015). Lead objects in their gizzards are mechanically slowly ground down and exposed to acids engaged in the digestion processes. In this way, the soluble lead salts are formed which can be absorbed into the blood stream. When present in blood, lead principally shows affinity to red blood cells (Katavolos *et al*., 2007). Consequently, if the concentration of lead is high enough, non-regenerative anaemia can be induced, specifically by impairing enzymes (d-aminolevulinic acid dehydratase (d-ALAD) and ferrochelatase) finally interfering with heme synthesis as well as by reducing the life span of erythrocytes (Levengood *et al*., 2000; Fair *et al*., 2007; Haig *et al*., 2014; Ferreyra *et al*., 2015). This physiological state can be manifested by a marked reduction of haematocrit. According to Campbell (1994), based upon studies of caged birds, decreases in haematocrit below 35% can be considered as a signal of progressing anaemia, while values between 35 and 55% are thought to be normal. Although haematocrit ranges were established for a limited number of wild bird species (Fair *et al*., 2007), it can be assumed that any declines in haematocrit, even within the normal range, can be evidence for effects of specific ecotoxic factors (i.e. lead exposure).

Lead may be relatively easily distributed via the circulatory system to the whole body of bird, and, subsequently, it accumulates in tissues and organs, leading to toxic effects. The most visible signs of such intoxication include weakness, anorexia and ataxia. Generally, this may be caused by damage to the nervous system or by paralysis of the digestive tract that impairs the digestion of food. Consequently, lead-intoxicated individuals have visible flying and walking difficulties, show weight loss, are more susceptible to predation and suffer from the occurrence of vomiting or intense greenish diarrhea which is a typical sign of advanced plumbism in birds (Day *et al*., 2003; De Francisco *et al*., 2003; Müller *et al*., 2008; Haig *et al*., 2014). Furthermore, lead has been recognized as an influential factor which can induce immunosuppressive effects. As a result, increased susceptibility to viral, bacterial and parasitic infections can be observed, which in consequence may reduce bird survival (Ferreyra *et al*., 2015). According to some studies (Eeva *et al*., 2005; Cid *et al*., 2018), the assessment of leukocyte profiles, in particular the heterophil-to-lymphocyte ratio (H/L ratio), can provide some feedback on heavy metal pollution, including lead.

Studying nestlings gives a special opportunity to gather data about the level of contamination and consequences of intoxication in exposed individuals in almost any area of interest (Markowski *et al*., 2014). Most studies performed on nestling great tits *Parus major* with respect to the possible effects of heavy metals on general health condition (Eeva and Lehikoinen, 1996; Dauwe *et al*., 2003) as well as morphometric and haematological indices (Eeva *et al*., 2000, 2009), were conducted in areas described as highly contaminated (Eeva and Lehikoinen, 1996; Dauwe *et al*., 2000). In such study systems, experimental treatments were applied at the ecological background where birds were chronically exposed to various trace elements. On the other hand, the number of similar studies conducted in areas recognized as unpolluted or described as those without clear sources of pollution is limited. In this kind of studies, birds can be rather exposed on accidental and short-term contamination. Consequently, as episodic exposures may be quite frequent and very important in urban conditions, they should be investigated experimentally.

Experimental studies on nestling great tits that used a single exposure to a single selected trace element are scarce. We are aware of a few studies in which lead was administrated to simulate exposure over a few days and to examine potential negative effects on nestlings (Eeva *et al*., 2014; Rainio *et al*., 2015; Ruuskanen *et al*., 2015; Ruiz *et al*., 2016). We know of no experimental study on physiological, morphological or behavioural alterations in nestling great tits in response to a single exposure to a heavy metal. Consequently, the aim of this study was to examine whether various levels of lead in the diet of the nestlings would result in any alteration of selected physiological indices, haematocrit level and H/L ratio. For this purpose, we conducted an experiment on nestling great tits, based on a single supplementation of lead acetate solution, with two dose levels.

## Materials and methods

This study was performed in 2016 as part of a long-term research project on the breeding biology of hole-nesting passerines in central Poland (Bańbura *et al*., 2007).

**Table 1:** Sample sizes, shown as the number of great tit nestlings for which heamatocrit and heterophil-to-lymphocyte ratio were examined, with regards to the dose and the age at which nestlings were supplemented with lead

	Dose of lead (μg/g of body mass)					
Age of lead exposure	Control	15	30	Control	15	30
	Haematocrit			H/L ratio		
9th	4	4	4	4	4	4
10th	9	10	10	9	9	10
11th	4	4	4	4	4	4

**Table 2:** Summary of a linear mixed model examining the level of haematocrit (%) in relation to the lead-supplementation experiment, time of exposure and body mass. Brood ID and study site were included as random effects

Factor (covariate)	df	*F*	*P*
Intercept	1;48.00	46.48	<0.0001
Lead dose	2;48.00	4.10	0.02
Age of lead exposure (cov)	1;48.00	12.93	0.001
Body mass (cov)	1;48.00	0.75	0.39
Removed non-significant interactions			
Lead dose^*^ age of lead exposure	2;41.00	0.89	0.42
Lead dose^*^ body mass	2;41.00	1.12	0.34
Age of lead exposure^*^ body mass	1;41.00	0.30	0.59

**Figure 1:**
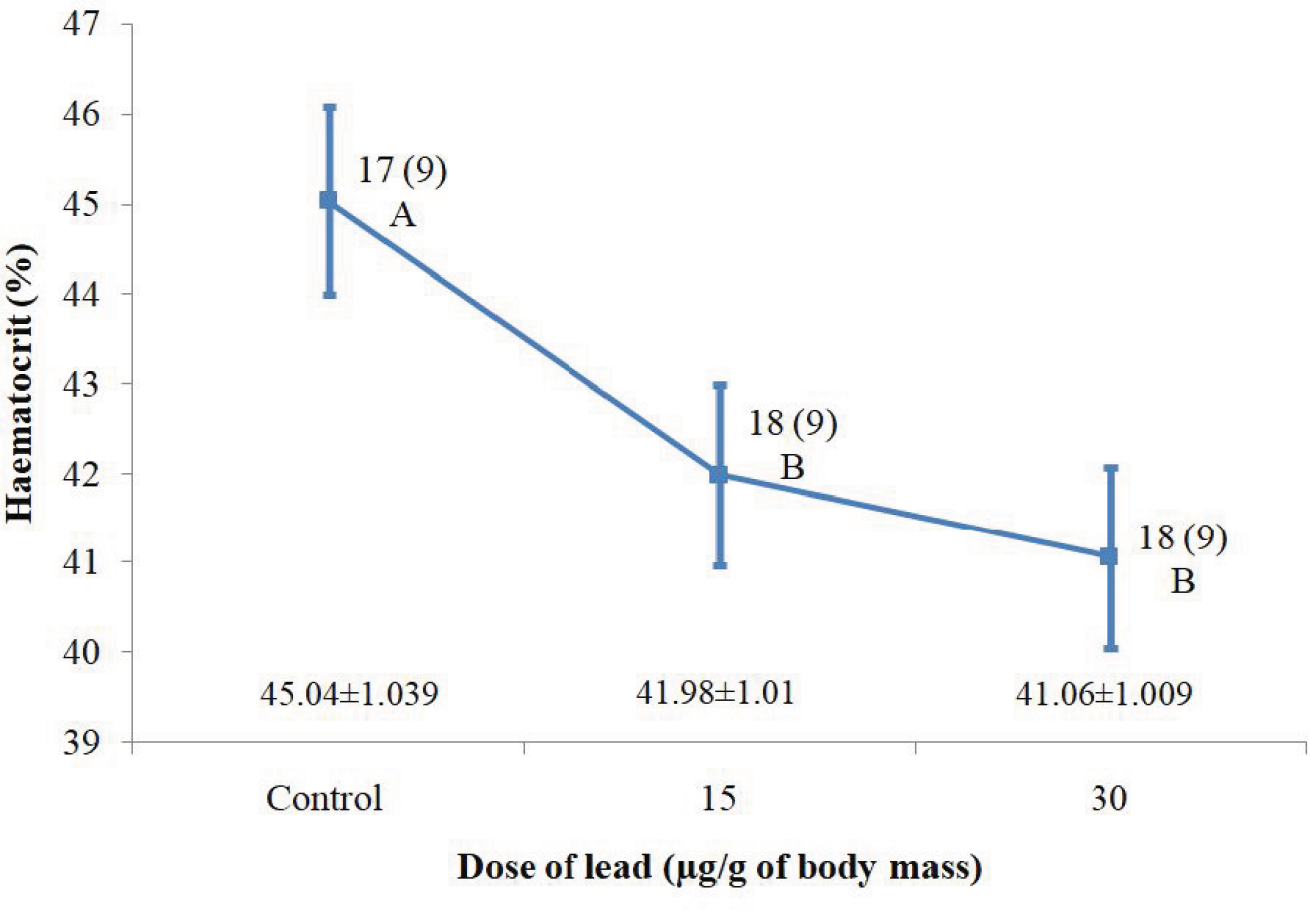
Haematocrit variation of great tit nestlings that were experimentally supplemented with lead acetate solution in comparison with control individuals. Mean ± standard errors calculated from the linear mixed model are shown. Sample sizes are shown as the number of nestlings and, in parentheses, as the number of broods. Differences among control and treatment groups using a Fisher’s LSD test are shown by letters ‘A’ and ‘B’. Means with the same letter indicate a non-significant test result

## Study sites

Study sites were established in two habitats—an urban parkland and a mature deciduous forest located in and around the city of Łódź. The urban parkland study site is situated in the southwest part of Łódź (51°45′N, 19°24′E) and encompasses the Łódź botanical and zoological gardens, covering ca. 80 ha. This area is characterized by a highly fragmented arrangement of vegetation often planted artificially and by intensive human activity (Markowski *et al*., 2014). The forest study site is located in the interior of the Łagiewniki Forest (51°50′N, 19°29′E) at the northeast border of Łódź and covers ~140 ha. Tree stands at the site consist mainly of mature deciduous trees of various species, with oaks *Quercus robur* and *Quercus petraea* being the predominant tree species (Bańbura *et al*., 2007; Markowski *et al*., 2013).

## Experiment

During the 2016 breeding season, an experiment on 11 randomly drawn broods of great tit from both study sites (six in the parkland area and five in the forest) was conducted. Regular inspections of wooden nestboxes were conducted starting from April, to record nesting bird species and, then, basic breeding traits (laying date, clutch size, brood size at the moment of hatching and the number of fledglings) (Markowski *et al*., 2015). In each brood, we assigned two randomly drawn nestlings to each of two lead-treatment groups (four nestlings in total), supplemented with two specified doses of lead. The first group included nestlings which received through the gape lead acetate solution at the dose of 15 μg per gram of their body weight, whereas the second group received a dose of 30 μg per gram of their body weight. From the remaining non-supplemented individuals in the nest, two nestlings were randomly drawn to be treated as a control group and received deionized water. On the day of lead exposure, nestlings from the supplemented groups were individually marked by nail cutting and weighed in order to calculate a proper dose of lead. Lead was administered orally as a lead II acetate trihydrate solution diluted in deionized water. The doses of lead used in this study were determined on the basis of our previous research (Markowski *et al*., 2013) and following the relevant literature (Eeva *et al*., 2014). In most of the papers, relevant variation in experimental exposure to lead is noticeable. Similarly to the study by Eeva *et al*. (2014), our intention was to simulate various levels of lead exposure, but with the use of a single dose per individual. The doses of 30 and 15 μg Pb/g of body mass used in this study correspond to lead burdens found at heavily polluted and less-polluted areas (Eeva *et al*., 2014). Supplementation procedures on nestlings (single application of lead per individual) were conducted on one of three days, the 9th, 10th or 11th day after hatching (with hatching day considered as Day 1) depending on the brood.

**Figure 2:**
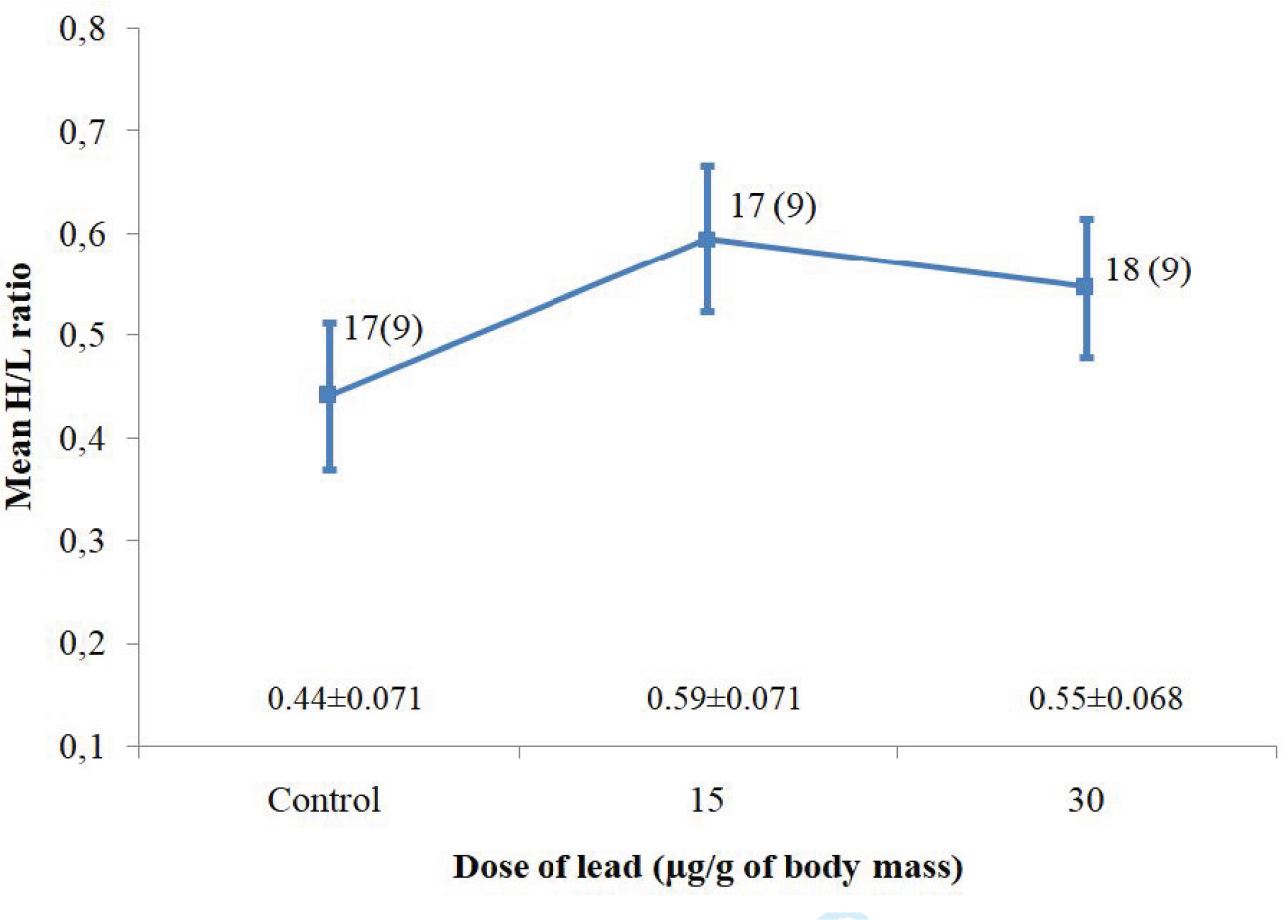
Mean H/L ratio variation of great tit nestlings that were experimentally supplemented with lead acetate solution in comparison with control individuals. Mean ± standard errors calculated from the linear mixed model are shown. Sample sizes are shown as the number of nestlings and, in parentheses, as the number of broods

The nestlings from both the treatment and control groups were individually banded with uniquely numbered metal rings and measured (wing length and body mass) on the 15th day after hatching. At the same time, blood samples were collected from the ulnar vein directly to 19 μL heparinised microcapillary tubes. Afterwards, microcapillary tubes with blood were stored in a cooler until processing and determining the value of haematocrit (%) in the laboratory (see details in Markowski *et al*., 2015). From the same individuals, additional blood samples were collected and transferred to a slide to prepare a blood smear. The smears were air-dried in the field and stained in the laboratory using a commercial Microscopy Hemacolor kit (Merck Chemicals). Subsequently, from each blood smear a random sample of 100 leukocytes was counted, applying a light microscope at ×1000 magnification with oil immersion. Identified white blood cells were classified into five cell categories: heterophils, lymphocytes, eosinophils, basophils and monocytes. The H/L ratio was calculated by dividing the number of heterophils by the number of lymphocytes. All microscope slides were analyzed by the same person (M.M.) in order to reduce inter-observer variability. To estimate repeatability of the H/L ratio, counts were conducted twice for each sample. Intra-class correlation coefficient (ICC) values indicate that repeatability of measurements was high (ICC = 0.83; *P* < 0.0001).

**Table 3:** Summary of a linear mixed model examining the H/L ratio in relation to the lead-supplementation experiment, time of exposure and body mass. Individual nestling ID, brood ID and study site were included as random effects

Factor (covariate)	df	*F*	*P*
Intercept	1;46.00	7.59	0.008
Lead dose	2;46.00	1.25	0.30
Age of lead exposure (cov)	1;46.00	9.05	0.004
Body mass (cov)	1;46.00	7.12	0.01
Age of lead exposure^*^ body mass	1;46.00	8.18	0.006
Removed non-significant interactions			
Lead dose^*^ age of lead exposure	2;40.00	0.06	0.94
Lead dose*body mass	2;40.00	0.14	0.87

Because of unexpected technical problems, such as microcapillary damage and non-readable smears, we finally measured haematocrit in 53 blood samples and analyzed blood smears for 52 nestlings from 9 broods, not in all cases from the same broods. Specific sample sizes are shown in Table 1.

## Statistical analysis

The effects of lead supplementation on haematocrit and H/L ratio were analyzed separately in relation to the dose category of lead acetate solution as a factor (three categories: control, low and high) by applying linear mixed models. The age at which nestlings were supplemented with lead and body mass were included in the model as covariates to control timing and developmental stage differences, respectively (Crawley, 2002). Brood ID and study site were included as random effects controlling for clustering, with degrees of freedom being approximated by the Satterthwaite method (Heck *et al*., 2010). Because leukocyte profiles were determined twice for each bird, in the mixed model related to H/L ratio we added individual nestling ID as another random factor to control the effect of non-independence for nestlings included in the experiment. Prior to statistical analyses, the individual H/L ratios were ln-transformed (ln (1 + H/L)) in order to normalize the distribution and stabilize the variance (Sokal and Rohlf, 1981). Both initial linear mixed models included the second-order interactions between all independent variables. Subsequently, non-significant interactions were removed to leave the final model that included only significant interactions and all independent variables (Crawley, 2002). Statistical analyses were performed using IBM SPSS 22 software (IBM SPSS Statistics 22, 2013).

## Results

We found that both of the dosages of lead applied in the experiment caused significant alterations in haematocrit levels (Table 2). Mean haematocrit values were lower in the treatment groups compared to the control groups, wherein the nestlings which received the highest dose of lead characterized the lowest mean haematocrit (Fig. 1). A Fisher’s LSD test showed significant differences between individuals from control and supplemented groups (control vs. 15 μg Pb/g of body mass *P* = 0.04; control vs. 30 μg Pb/g of body mass *P* = 0.008). No significant differences were found between the two levels of treatment (15 μg Pb/g of body mass vs. 30 μg Pb/g of body mass, *P* = 0.52).

Furthermore, the day of exposure to lead in the middle of the postnatal period significantly affected haematocrit variation in nestling great tits. A decrease in haematocrit was observed on the consecutive days of the nestlings’ lives (9–11) on which lead administration was performed (Table 2, effect estimate −3.15 ± 0.88). This means that the later lead was administered to a nestling, the lower was the value of its haematocrit on the 15th day after hatching. At the same time, body mass revealed no significant effect on haematocrit (Table 2).

The comparison of the H/L ratio between nestlings from both the treatment and control groups showed a non-significant difference (Table 3, Figure 2). Both age of lead exposure and body mass positively affected the H/L ratio (effect estimate 2.40 ± 0.80 and 1.35 ± 0.51 respectively), but they mutually modified their effect, resulting in a significant interaction (Table 3).

## Discussion

In this study, we found that the supplementation of two different amounts of lead compounds in the diet of great tit nestlings, even when in the form of one-off dose, results in a significant haematocrit decrease (Fig. 1). The day on which experimental exposition to lead occurred turned out to be another significant predictor of changes in haematocrit. This is useful information on physiological responses to a heavy metal exposure of small passerines; in this particular case, lead exposition affected the level of haematocrit. Such experimental studies performed on great tit nestlings are scarce and to our current knowledge were only conducted in south-western Finland (Eeva *et al*., 2014; Rainio *et al*., 2015; Ruuskanen *et al*., 2015; Ruiz *et al*., 2016). In contrast to our results, Eeva *et al*. (2014) did not find significant differences in haematocrit level among three experimental groups of nestlings (control, low and high Pb dosed). Moreover, the values of haematocrit recorded by Eeva *et al*. (2014) were on average lower for control and supplemented groups than those found in our study (ranged from ca. 38–40 and 41–45%, respectively) and did not indicate any coherent pattern, in contrast to our findings which found a clear tendency to decrease. These discrepancies in the results seem to be connected with substantial differences in the methodology applied, which means that direct comparisons should be considered with care. First of all, in our study, we decided to simulate an exposure of nestlings to two various single doses of lead on Days 9–11 of their life, while Eeva *et al*. (2014) exposed nestlings to lead for 12 days starting from the 3rd and up to the 14th day of their life. Secondly, in either study blood samples were collected at different age of the nestlings—Eeva *et al*. (2014) collected blood samples to determine haematocrit on Day 7 of nestling life, whereas it was done on Day 15 in our study. It is well known that some haematological parameters, including haematocrit, undergo significant changes during nestling growth and development, tending to increase with age (Kostelecka-Myrcha *et al*., 1973; Kostelecka-Myrcha and Jaroszewicz, 1993; Kostelecka-Myrcha *et al*., 1997; Dawson and Bortolotti, 1997; Kostelecka-Myrcha and Chołostiakow-Gromek, 2001; Gayathri *et al*., 2004; Simmons and Lill, 2006; Kaminski *et al*., 2014). According to Kostelecka-Myrcha *et al*. (1973), during the nestling development of great tits, haematocrit clearly increases intensively in the first 8 days of life and then still further, but at a slightly slower pace, up to reaching a stable level just before the fledging time. Kostelecka-Myrcha *et al*. (1973) showed that on the 7th and 15th days haematocrit in nestling great tits was ca. 35% and 43% of packed red blood cells in whole blood, respectively, which corresponds to the outcomes reported by Eeva *et al*. (2014) and those presented in our study.

It is conceivable that in the very early nestling phase, in which the nestlings were supplemented by Eeva *et al*. (2014), the mechanism of rapid substitution of injured erythrocytes could be activated (Hernández-García *et al*., 2014). Presumably, this could help to balance changes in blood characteristics, which could result in non-significant variation in haematocrit level as stated by Eeva *et al*. (2014), despite the exposure to lead. Additionally, despite the chronic exposure, till the day of haematocrit evaluation, the nestlings received lead just for 4 days, which in fact gave relatively low total doses (4 and 16 μg Pb/g of body mass, respectively for low and high groups of treatment).

By contrast, our experimental nestlings were more advanced in age. In older nestlings, blood parameters become more stable and when lead appears in the bloodstream and impairs the function of its components, achieving homeostasis is more difficult in comparison with the initial period of haematopoietic ontogenesis. To confirm this explanation, once again one should refer to significance of the age of exposure. We found that older nestlings which received lead characterized more declined haematocrit levels.

Other experimental studies have also confirmed that the ingested lead was responsible for a significant decrease in haematocrit. This was recorded by Cid *et al*. (2018) in a study conducted on House sparrows *Passer domesticus*, but only at the highest doses of exposure (>7 μg of Pb/g/day). Similar results were presented by Plautz *et al*. (2011) who stated that lead dosed Mourning doves *Zenaida macroura* for a few days exhibited greater alterations in haematocrit in comparison to non-exposed birds, decrease up to ca. 38% in comparison to 44%, respectively. Comparable conclusions were drawn in a study performed on American kestrel *Falco sparverius* nestlings which were lead-supplemented for several days (Hoffman *et al*., 1985) as well as on mute swans cygnets exposed to lead for several weeks (Day *et al*., 2003). Hoffman *et al*. (1985) revealed that after 10 days, in the highest lead dosed groups of American kestrel nestlings which received 125 and 625 mg of Pb/kg of body mass, haematocrit values were significantly lower (29.3 and 23.4%, respectively) than in control individuals (34.1%). In the case of study conducted by Day *et al*. (2003), mute swans cygnets that were exposed to the highest doses of lead (3.9 and 7.9 μg of Pb/g of body mass) had also significantly reduced haematocrit (40.9 and 43.6%, respectively) in comparison to control individuals (47.9 and 51.8, respectively). However, most such studies were conducted on raptors, scavengers and waterfowl, exposed in the polluted environment. In the majority of such papers, it was consistently found that birds which consumed lead had lower haematocrit (Carpenter *et al*., 2003; De Francisco *et al*., 2003; Pattee *et al*., 2006; Katavolos *et al*., 2007; Haig *et al*., 2014; Ferreyra *et al*., 2015).

Because heamatocrit in wild birds can reduce under the impact of various environmental stressors, both natural and anthropogenic (Fair et al. 2007), a possible stress related to capture and handling can be considered as one of the determinants of variation.

In this study, the handling stress associated with application activity was the same for all lead/control treatment groups and lasted no longer than 10 min. Therefore, we think that this action could not contribute to significant differences in haematocrit. Similar findings were presented by D’Amico *et al*. (2017) in the study performed on two-banded plovers *Charadrius falklandicus*. The authors found that heamatocrit showed no changes over the broad range of handling time, up to 232 min.

Amongst many physiological blood parameters, haematocrit has been frequently suggested to be a potentially valuable indicator of physiological condition. However, its validity has been recently disputed (Fair *et al*., 2007; Minias, 2015). Haematocrit is a blood parameter influenced by various kinds of factors, such as age, sex, geographical elevation, energy expenditure, parasitism, nutrition and heritability (Fair *et al*., 2007). Therefore, according to Fair *et al*. (2007) and Minias (2015), it should not be recommended to use as a sole indicator to evaluate general condition of birds. Nevertheless, despite these doubts, the assessment of haematocrit is pointed out as one of the best methods to diagnose anaemia in birds (Fair *et al*., 2007) and, in this regard, it can be treated as a simple indirect method to evaluate lead intoxication. This is confirmed by our results as well as by other authors (Hoffman *et al*., 1985; Miller *et al*., 2001; Carpenter *et al*., 2003; Day *et al*., 2003; De Francisco *et al*., 2003; Pattee *et al*., 2006; Katavolos *et al*., 2007; Haig *et al*., 2014; Ferreyra *et al*., 2015). However, we suggest that particular attention should be paid to the methodology used. Especially, it would be essential to collect blood samples on the properly standardized day of nestling life. This would avoid methodological difficulties and effects of various influential factors, for instance, the dynamic development of the respiratory function of blood volume unit in nestlings, visible in many birds, especially altricial species (Kostelecka-Myrcha and Jaroszewicz, 1993). We suggest that for great tit nestlings, blood sampling and haematocrit examination should be conducted at ca. 15th day of their life. This results from the fact that in great tit nestling development, two various periods of haematocrit increase can be distinguished. While the first 8 days of their life characterize significantly higher rates of haematocrit rise, the consecutive days are less dynamic in this respect (Kostelecka-Myrcha *et al*., 1973). Consequently, at the 15th day of great tit nestling life, haematocrit reaches a relatively stable level, which gives an opportunity to consider effects of various external factors on its variation (i.e. lead exposure).

Exposure to heavy metals has been reported as a considerable environmental stressor for animals. In this regard, the measurement of the H/L ratio is thought to be a practical tool to evaluate physiological stress in birds (Eeva *et al*., 2005; Cid *et al*., 2018). In our study, we showed that a single exposure to an increased dose of lead did not affect the H/L ratio of great tit nestlings in comparison to non-exposed individuals (Table 2). This can be explained by the fact that in such a short-term condition of lead intoxication, regardless on a dose used, the immune system is not capable of inducing a rapid response, manifested in a detectable change in the leukocyte profile. Additionally, we cannot entirely exclude the possibility that the size of the sample we had could limit the power of statistical tests. However, as was shown in other experimental studies, regardless of the sample size used, the outcomes concerning the H/L ratio altered by lead exposure are much the same as those presented in this paper. For instance, a similar conclusion can be drawn from the findings presented by Cid *et al*. (2018), who proved in an experimental study that the acute and chronic lead exposure of house sparrows, leads to different H/L ratio responses. Cid *et al*. (2018) found that the H/L ratio did not differ significantly in birds exposed to lead for a short period of time (5 days of treatment) in comparison to non-exposed individuals while lead exposure for longer periods (15 and 30 days of treatment) resulted in a significant increase of H/L ratio. Similar results were obtained in the study on mourning doves where the H/L ratios recorded were significantly higher after a few days of lead exposure compared to non-exposed individuals (Plautz *et al*., 2011). Apparently, in the case of lead poisoning, the H/L ratio can be considered as a biological indicator of chronic stress that develops over the longer term. Furthermore, this confirms the earlier findings that elevated level of the H/L ratio provides information about long-term effects of the stress factors (Davis *et al*. 2008; Bańbura *et al*., 2013). However, one should notice that in the case of lead, not only the time of exposure determines the H/L ratio. In another experimental study, Grasman and Scanlon (1995) demonstrated that food may be an important factor which could enhance the toxic effect of lead. They found elevated H/L ratios in the lead exposed groups of Japanese quail *Coturnix coturnix* which were fed with corn and reduced ratios in lead-exposed individuals which were fed with poultry food. This suggests that in evaluation of any alteration in H/L ratio induced by lead, various additional factors should be considered as relevant ones. Certainly, in experimental studies, handling stress associated with application of the lead/control treatment to birds should be analyzed with caution. This results from the fact that in general H/L changes may be species specific. For Adelie penguins *Pygoscelis adeliae* (Vleck *et al*. 2000) and house finches *Carpodacus mexicanus* (Davis 2005), handling time did not affect the H/L ratio up to 1 h, while for wintering male great tits this caused an increase in number of heterophil between 30 and 60 min and a decline in lymphocyte number between 60 and 120 min after capture (Cïrule *et al*. 2012). Also, O’Dell *et al*. (2014) studying tufted titmouse *Baeolophus bicolor* recorded that H/L ratio was not affected by handling time. In our study, all experimental procedures took no more than 10 min per each treatment group in broods; therefore, we claim that this factor could not play a crucial role in H/L ratio variation.

In conclusion, this study suggests that physiological effects of a single lead exposure are indicated by changing values of haematocrit. We found that haematocrit in great tit nestlings significantly declined after the exposure to increasing single doses of lead. At the same time, this type of lead exposure did not affect the H/L ratios. In accordance with some other studies, we think that the H/L ratio is a more appropriate index to use in evaluation of chronic lead exposure and chronic stress, in general.

## Ethics statement

All performed procedures in the study were approved by the Local Ethical Committee and the State Office for Environment Protection in Łódź (WPN-II.6401.13.2016.MS, WPN-II.6401.122.2016.KW2).

## Conflict of interest

The authors declare that there are no conflicts of interest.
